# AI literacy as both bridge and buffer: unraveling its dual role between research stressors and teaching excellence

**DOI:** 10.3389/fpsyg.2026.1758306

**Published:** 2026-05-07

**Authors:** Guimei Yang, Feng Liu, Qingjie Zhao

**Affiliations:** 1 School of Business, Fuyang Normal University, Fuyang, China; 2 School of Management, Sichuan University of Science and Engineering, Zigong, China

**Keywords:** artificial intelligence literacy, challenge-hindrance research stressors, dynamic capabilities, generative artificial intelligence, sustainable higher education, teaching excellence

## Abstract

**Introduction:**

The persistent tension between research and teaching poses a significant challenge to sustainable development in higher education. Grounded in the Challenge-Hindrance Stressors Framework and Dynamic Capabilities Theory, this study investigates the dual role of faculty artificial intelligence (AI) literacy, conceptualized as a dynamic capability to sense, seize, and reconfigure AI resources to reshape the impact of research stressors on teaching excellence.

**Methods:**

We conducted a survey of 253 faculty members from Chinese universities. The proposed model, which delineates the asymmetric effects of challenge and hindrance research stressors, the mediating role of teaching-research time conflict, and the dual mechanisms of AI literacy, was tested using partial least squares structural equation modeling.

**Results:**

Challenge research stressors significantly enhanced teaching excellence, both directly and indirectly through increased AI literacy. Conversely, hindrance stressors exacerbated teaching-research time conflict but did not exhibit a direct negative effect on teaching excellence. Notably, AI literacy demonstrated a dual mechanism: it mediated the positive effect of challenge stressors and also buffered the negative impact of teaching-research time conflict on teaching excellence.

**Discussion:**

These findings suggest that when AI literacy functions as a dynamic capability, the competitive relationship between research and teaching can become synergistic. The study extends the Challenge-Hindrance Stressors Framework by showing that different types of research pressure have distinct implications for teaching outcomes. It also contextualizes the Conservation of Resources Theory by identifying AI literacy as a boundary condition: in its presence, the conventional mechanism of resource drain is disrupted. By reframing AI literacy as a capability for resource reconfiguration rather than merely a technical skill, this research suggests a potential direction for institutions: moving from mitigating research pressure to leveraging generative AI for faculty development. Strengthening AI literacy may therefore serve as a practical lever for advancing student-centered education and the broader sustainability agenda.

## 1 Introduction

Against the backdrop of global higher education’s commitment to achieving the United Nations Sustainable Development Goals, continuous improvement of teaching quality has become a central issue (Vykydal et al., 2020; UNESCO, 2021). High-quality education is not only a goal for sustainable development, but also the foundation for achieving all other goals (United Nations, 2015; Abera, 2023). A profound paradox persists in higher education. The global academic system remains entrenched in a “research-first” paradigm that, through performance evaluation and ranking mechanisms, systematically crowds out investment in teaching, forming an unsustainable trend of “sacrificing teaching for research” (Tian and Lu, 2017; Li and Zhang, 2024). This global challenge serves as the starting point for this study’s exploration of how to leverage intelligent technology to reshape teaching excellence and realize the vision of sustainable educational development.

This global challenge manifests with particular specificity and depth in the context of Chinese higher education’s journey towards “connotative development.” To address the core issues of “insufficient innovation and lack of challenge” in undergraduate education and respond to the urgent demand for top-notch innovative talent under the national innovation-driven development strategy, the Chinese Ministry of Education launched the landmark “Golden Course Construction Plan” in 2019. This initiative aims to create high-quality courses characterized by high-order thinking, innovation, and challenge (Chinese Ministry of Education, 2019), a standard collectively known as “golden course quality (GCQ),” which establishes the official benchmark for teaching excellence in China’s higher education system. The plan is designed to catalyze a quality revolution in the undergraduate curriculum and to establish teaching innovation as a central pillar of high-quality development in higher education, thereby endowing faculty with a teaching mission that is equally important as their research mission.

However, within an academic ecosystem that prioritizes research excellence as the primary criterion for evaluation (Cadez et al., 2015; Tian and Lu, 2017), the dual missions of teaching innovation and research compete for faculty’s finite time and cognitive resources, creating profound tension. Reforms in China’s academic appointment system, notably the widespread adoption of the “tenure-track” model, have further intensified research pressure on early-career faculty (Si, 2023). Consequently, as faculty become increasingly absorbed into “publish or perish,” the deep instructional design required for GCQ is often compromised (Tian and Lu, 2017; Bello et al., 2023). A large-scale survey by Li and Zhang (2024) confirmed that research time significantly encroaches upon teaching time. Traditionally, this tension is viewed through a linear lens of “resource depletion,” whereby research pressure crowds out instructional time and thus diminishes teaching quality.

Conservation of Resources (COR) Theory epitomizes this traditional explanatory framework. Its core tenet holds that individuals are fundamentally motivated to acquire, retain, and protect valued resources (e.g., time, energy). When confronted with pressure threats that risk net resource loss, they adopt defensive behavioral strategies to prevent further resource depletion (Hobfoll, 1989). Within this framework, research pressure erodes teaching quality by exacerbating the teaching-research time conflict, a perceived state in which faculty are compelled to reduce teaching resource allocation to meet research demands. To preserve research resources perceived as delivering higher career returns, faculty reduce their teaching investment, thereby systematically impairing teaching quality (Lai et al., 2014).

However, the explosive development of generative artificial intelligence (GenAI) challenges the “absolute resource scarcity” assumption foundational to this theory. Recent studies suggested GenAI automates core teaching tasks (e.g., course design, personalized learning support, and intelligent assessment) to markedly reduce instructional preparation time (Chiu, 2023; Mulyani et al., 2025; Roy et al., 2024). More critically, through task automation, personalized feedback provision, and the generation of pedagogical insights, GenAI demonstrates strong potential to empower faculty to balance high-quality research with excellence in teaching practice (Ganguly et al., 2025). Whether faculty can translate the potential of GenAI into sustainable capacity to maintain and enhance teaching quality fundamentally depends on their AI literacy. Drawing on Dynamic Capability Theory (Teece, 2007), we conceptualize AI literacy as a dynamic capability encompassing three core competencies: sensing and identifying AI-enabled opportunities; seizing these opportunities through technical proficiency; and reconfiguring teaching resources through critical, analytical AI application to transform instructional practices. This implies that technological empowerment variables may reconfigure the traditional stress transmission pathway.

Despite this considerable potential, whether GenAI reshapes the transmission mechanism linking research pressure to GCQ remains unclear, constrained by three critical gaps:

First, there is a lack of differentiation regarding research stressor types. Existing literature has overlooked the heterogeneous effects of research pressure on teaching quality (Bak and Kim, 2015; Cadez et al., 2015). Organizational behavior research has established that challenge stressors foster innovation by enhancing cognitive flexibility, whereas hindrance stressors trigger resource depletion (Lee and David, 2022; Ma and Hu, 2025). However, this differentiated effect mechanism has not been extended to the teaching domain. This gap not only restricts policymakers’ ability to implement precise interventions targeting the sources of research pressure but may also impede GCQ improvement due to misjudgments of stressor attributes. Clarifying this heterogeneity is also a fundamental theoretical prerequisite for evaluating the differentiated empowering effects of GenAI.

Second, empirical support for the mediating role of teaching-research time conflict is lacking. Although COR positions teaching-research time conflict as the core mediating variable, rigorous quantitative evidence is severely scarce, with existing conclusions primarily derived from qualitative interviews (Lai et al., 2014; Tian and Lu, 2017; McCune, 2019). Empirical testing of the mediating effects in the stress transmission mechanism is critical in both workplace and educational settings (Buzás et al., 2025; Shang et al., 2025). No large-sample studies have validated the effect size and statistical significance of teaching-research time conflict in the “research pressure → GCQ” pathway, let alone explored its heterogeneous patterns under challenge versus hindrance stressors.

Most critically, the technological empowerment pathway remains a dual black box. The efficacy of GenAI is highly dependent on the faculty’s AI literacy (Dwivedi et al., 2023). We theoretically posit that AI literacy plays a dual role amid the tension between universities’ dual missions of teaching and research. It acts as an empowering mediator that translates research pressure into GCQ, and as a buffering factor that mitigates the negative impact of teaching-research time conflict on teaching quality. Specifically, two core questions remain unaddressed. (1) Can research pressure indirectly optimize GCQ by enhancing AI literacy? (2) Can high AI literacy buffer the constraining effect of teaching-research time conflict on GCQ? To the best of our knowledge, no prior study has empirically examined this dual mechanism.

To address the aforementioned theoretical gaps, this study proposes the following core research questions:

*RQ1*: In a research-oriented academic ecosystem, do challenge and hindrance research stressors exert asymmetric effects on GCQ?

*RQ2*: Does teaching-research time conflict serve as a significant mediating role in the relationships between the two types of research stressors and GCQ?

*RQ3*: Does AI literacy play a dual role in the transmission mechanism linking research stressors to GCQ?

*RQ3a*: Does AI literacy mediate the "research stressors → GCQ" pathway?

*RQ3b*: Does AI literacy moderate the inhibitory effect of teaching-research time conflict on GCQ?

The primary value of this study lies in its contribution to sustainable education. Theoretically, by empirically testing the asymmetric effects of challenge and hindrance research stressors on GCQ and revealing the dual role of faculty AI literacy in the research stress transmission pathway, this study not only challenges the linear perception that “research pressure inevitably harms teaching”, but more crucially, extends COR Theory by identifying boundary conditions under which its assumptions operate differently in AI-enabled academic environments. It also offers a new perspective on how faculty navigate dual-mission tensions in the digital-intelligence era. At the policy level, the findings provide empirical evidence and a technology-enhanced pathway for higher education institutions to optimize research pressure management, deeply integrate GenAI tools into faculty development systems, and strengthen AI literacy training. This supports the implementation of the “student-centered education” philosophy and advances the national strategy for cultivating top-tier innovative talent, ultimately contributing to the sustainable development of higher education.

## 2 Literature and hypotheses

### 2.1 Golden course quality: a policy-driven construct of teaching excellence

GCQ serves as the official benchmark for teaching excellence in China’s higher education system, as established by the national “Golden Course Construction Plan” (Chinese Ministry of Education, 2019). It is conceptualized as the extent to which undergraduate courses achieve integrated excellence across three core dimensions, namely high-order thinking (the integration of knowledge, skills, and qualities, aiming to cultivate students’ comprehensive ability to solve complex problems and advanced thinking), innovation (the incorporation of frontier academic content or innovative pedagogical methods), and challenge level (coursework with appropriate rigorous learning tasks that requires students to stretch their abilities, placing high demands on both teachers’ instructional preparation and students’ after-class learning effort).

While these dimensions originate from a specific policy context, they reflect universal concerns in educational psychology and higher education research. High-order thinking captures the core emphasis of deep learning theorists: meaningful education moves beyond rote memorization toward the ability to apply, transfer, and synthesize knowledge across contexts (Biggs, 1987; Entwistle and Ramsden, 2015). When faculty design courses that require students to analyze, evaluate, and create, they engage in pedagogical transformation, translating their own research insights into learning experiences that mirror the complexity of real-world problems, i.e., a mechanism further supported by experiential learning theory (Kolb, 2014).

Innovation reflects the dynamic nature of knowledge itself. As Fullan (2005) argued, curriculum responsiveness is not an optional enhancement but a fundamental requirement for education to remain relevant to evolving disciplinary landscapes, while the TPACK framework specifies how technological and pedagogical innovations must be integrated with content knowledge to produce meaningful learning outcomes (Koehler and Mishra, 2009).

Challenge level is grounded in cognitive load theory and deliberate practice theory. Cognitive load theory holds that learning is most effective when tasks are sufficiently demanding to engage deeper cognitive processing without overwhelming working memory (Sweller, 1988). The deliberate practice theory shows that sustained, effortful engagement beyond formal instructional time is essential for developing expertise (Ericsson et al., 1993).

The golden course framework defines the intrinsic pedagogical quality directly influenced by faculty’s research engagement and technological competence. Unlike traditional teaching effectiveness metrics focused on student satisfaction, course ratings, or test scores, GCQ emphasizes pedagogical dimensions most susceptible to research pressure. High-order thinking and innovation benefit from cutting-edge research and knowledge transfer. Challenge level may suffer when research consumes teaching time. This heterogeneity makes GCQ an ideal outcome variable for examining how different research stressors shape teaching outcomes.

### 2.2 Research stressors and golden course quality: based on the challenge-hindrance stressors framework

The Challenge-Hindrance Stressors Framework serves as the core theoretical foundation for analyzing the effects of research stressors on GCQ. According to Cavanaugh et al. (2000), this framework dichotomizes work stressors into challenge stressors and hindrance stressors. Challenge stressors stem from job demands directly aligned with core work goals. Despite their inherent difficulty, they are perceived as growth-facilitating, with the potential to drive motivation, competency development, and goal attainment. In contrast, hindrance stressors stem from contextual factors that impede goal achievement and professional growth, primarily depleting individual resources and inhibiting outcomes (Cavanaugh et al., 2000). Subsequent studies have widely validated and extended this framework, confirming its robust explanatory power in knowledge-intensive professional contexts, including higher education academia (Webster et al., 2011; Ma and Hu, 2025).

Within the research context, this study defines challenge research stressors as the pressure perceived by university faculty stemming from demanding tasks directly linked to their core research objectives. Although challenging, such pressure promotes cognitive flexibility (Lee and David, 2022) and strengthens intrinsic motivation (Ma and Hu, 2025) due to its inherent connection to goal achievement. By contrast, hindrance research stressors refer to pressure perceived by faculty as irrelevant or obstructive to core research goals, undermining research effectiveness and scholarly development (Ma and Hu, 2025; Pitafi et al., 2025). These stressors are viewed as impeding academic progress and valued goal achievement (Cavanaugh et al., 2000; Pitafi et al., 2025), as they continuously deplete limited temporal and psychological resources, ultimately triggering goal disengagement and burnout (Korunka et al., 2015).

Challenge research stressors are expected to enhance GCQ by facilitating knowledge transfer, prompting the faculty to actively integrate cutting-edge research findings, complex problem-solving paradigms, and methodological systems into curriculum design (Pitafi et al., 2025). This positive pathway has received empirical support in recent studies. Xu et al. (2024) found that challenge stressors positively influence innovative behavior among higher education teachers, with task crafting serving as a critical mediating mechanism. This finding suggests that challenge stressors activate adaptive cognitive strategies that enable faculty to restructure their work, a process that can extend across the research-to-teaching continuum. Similarly, Wu et al. (2024) demonstrated that challenge stressors influence innovative work behavior through the serial mediation of creative self-efficacy and creative motivation, highlighting the motivational pathways through which challenge stressors translate into pedagogical innovation.

The relationship between hindrance stressors and teaching outcomes is less straightforward. Prior research has documented mixed findings regarding the effects of hindrance stressors on performance outcomes. While the Challenge-Hindrance Stressors Framework traditionally predicts negative effects (Cavanaugh et al., 2000), some studies suggest that hindrance stressors may operate through indirect pathways rather than directly (LePine et al., 2005). Ma and Hu (2025) found that while challenge research stressors positively influenced researchers’ innovative behavior, hindrance stressors exerted their negative effects through the sequential mediating mechanisms of achievement motivation and research anxiety rather than through direct pathways. Beyond the resource-based perspective, scholarship on value conflict and academic identity offers alternative lenses. Drawing on value conflict theory, De Graaf (2021) demonstrated that academics facing tensions between teaching quality and external pressures, such as research demands or heavy teaching loads, often adopt hybridization strategies, actively reconciling competing values rather than passively accepting role conflicts. Similarly, Frings et al. (2024) found that identity-based incompatibilities between researcher and educator roles predicted mental health outcomes, whereas practical incompatibilities such as time conflict showed no direct links. Extending this logic, their findings suggest that identity-related factors may outweigh practical constraints in shaping academics’ responses to external pressures, a pattern that could plausibly extend to teaching outcomes. This resonates with McCune’s (2019) theory of academic identity agency, which posits that faculty actively protect valued aspects of their professional identity when facing external threats. In the Chinese context, where the “Golden Courses” policy has elevated teaching innovation to a strategic priority (Chinese Ministry of Education, 2019), such identity protection mechanisms may be particularly salient. Given these mixed findings, we propose the following hypotheses:

*H1a*: Challenge research stressors positively impact GCQ

*H1b*: Hindrance research stressors may negatively impact GCQ

### 2.3 Teaching-research time conflict: a conservation of resources theory-based mediating mechanism

Teaching-research time conflict reflects competition for limited temporal resources between research and teaching responsibilities. The concept aligns with COR Theory, which states that resources are absolutely scarce. Individuals are naturally inclined to acquire and protect valuable resources (Hobfoll, 1989, 2001; Westman et al., 2004). Research pressure triggers a reallocation of resources. Faculty prioritize research activities, since they regard research as generating higher career returns, thereby reducing their investment in teaching (Lai et al., 2014; Bak and Kim, 2015; Tian and Lu, 2017). In multitasking contexts, this leads to defensive teaching strategies, such as simplified course design and compressed high-order cognitive activities (Bak and Kim, 2015; Gilbert et al., 2021). Institutional forces worsen this conflict, and the “publish or perish” culture undervalues teaching. Promotion is largely driven by research reputation, while teaching innovation receives insufficient institutional support (Gilbert et al., 2021; Bello et al., 2023).

This process creates a clear theoretical pathway. Research stressors give rise to teaching-research time conflict, which in turn leads to simplification of teaching practices. Cross-national studies have confirmed this pattern (Lai et al., 2014; Bak and Kim, 2015). However, digital transformation may change this outcome. Digital tools enable faculty to restructure workflows and potentially mitigate the adverse effects of time conflict on GCQ (Chiu, 2023; Mulyani et al., 2025; Roy et al., 2024). Given this open question, the following exploratory hypotheses are proposed:

*H2a*: Challenge research stressors may influence GCQ through teaching-research time conflict.

*H2b*: Hindrance research stressors may influence GCQ through teaching-research time conflict.

### 2.4 The dual enabling mechanism of AI literacy: a dynamic capabilities perspective

The rise of GenAI represents a significant environmental transformation in higher education. This transformation requires a theoretical lens that moves beyond static resource-based perspectives (O’Dea, 2024). Dynamic Capabilities Theory (Teece, 2007) offers a valuable framework by focusing on how individuals develop capacities to adapt to rapid change, including sensing opportunities, seizing them through new tool adoption, and reconfiguring resources to enhance effectiveness. In the context of digital transformation, faculty dynamic capabilities manifest as three specific abilities: identifying AI’s potential, learning and applying GenAI tools, and integrating them into teaching and research practices.

Grounded in this perspective, we conceptualize AI literacy as a dynamic capability that enables individuals to quickly sense technological shifts, seize opportunities through learning and the integration of GenAI tools, and thereby reconfigure teaching and research practices to enhance adaptability and efficacy. This capability is essential for sustainable teaching practices in the digital era. It encompasses four dimensions: (1) AI basic: understanding technical principles (serves sensing); (2) AI proficiency: designing effective prompts (serves seizing); (3) AI insight: identifying technological limitations and ethical risks (mitigates risks, ensuring effective reconfiguration); (4) AI analytical capability: using AI to solve complex problems (serves seizing and reconfiguring).

This understanding of AI literacy engages with two interrelated strands of the recent literature. On the one hand, generative AI creates cognitive dissonance for users. Its efficiency gains often conflict with core academic values, such as originality and intellectual effort (Seran et al., 2025). On the other hand, AI literacy is a multidimensional capacity that encompasses technical understanding, proficient application of AI tools, critical evaluation of outputs, and the ability to address complex problems (Imjai et al., 2025). These dimensions form a capability framework that enables faculty to navigate the inherent tensions of AI integration. This framework supports faculty in sensing technological shifts and underlying value conflicts. They seize tools with ethical awareness and reconfigure teaching practices to balance efficiency with academic integrity. The framework reframes AI literacy not merely as a technical skill, but as a comprehensive capacity to integrate technology with academic values.

Drawing on Dynamic Capabilities Theory (Teece, 2007), AI literacy plays a dual role in the pathway from research stressors to GCQ. First, it mediates the effects of different types of research stressors on GCQ. Second, it buffers the negative impact of teaching-research time conflict on GCQ.

Regarding its mediating role, challenge research stressors typically stimulate faculty’s learning motivation and adaptive behaviors (Cavanaugh et al., 2000; Ma and Hu, 2025). In the context of the rapid development of GenAI, this pressure drives the development of AI literacy. Faculty deepen their understanding of large model principles (AI basics, serving sensing). They systematically practice complex prompts to accurately obtain desired content (AI proficiency, serving seizing). They critically assess the reliability and ethical boundaries of the generated outputs (AI insights, mitigating risks for effective reconfiguration). Moreover, they explore novel methods for AI-assisted research, such as data analysis and literature review (corresponding to AI analytical capability, serving seizing and reconfiguring) (Chiu, 2023; Ji et al., 2025; Imjai et al., 2025). This enhanced AI literacy directly contributes to the three dimensions of GCQ. Faculty use AI analytical capability to design inquiry-based projects that foster high-order thinking. They leverage AI capabilities to generate novel cases and personalized learning pathways that foster innovation. Additionally, they build on AI knowledge to design progressively more challenging tasks. This process embodies the dynamic capability cycle: faculty sense technological changes, seize opportunities by learning GenAI tools, and reconfigure their teaching content and methods accordingly.

Conversely, hindrance research stressors deplete faculty’s psychological resources and cognitive bandwidth, dampening their willingness to explore and apply new technologies (LePine et al., 2005). Such suppression leads to stagnated development or insufficient application of AI literacy. It hinders the faculty’s dynamic capability to use GenAI for innovation and cognitive deepening in teaching (Sun et al., 2023). Consequently, GCQ is indirectly impaired.

Furthermore, as a dynamic capability, AI literacy plays a crucial buffering role. Teaching-research time conflict represents a classic scenario of resource scarcity that typically undermines teaching quality (Lai et al., 2014; Bak and Kim, 2015; Tian and Lu, 2017). However, faculty with high levels of AI literacy can effectively reconfigure resources and processes through their dynamic capabilities. They apply AI proficiency to streamline the generation of teaching materials, use AI analytical capabilities to redesign student progress diagnostics to improve efficiency, and utilize AI insights to proactively identify and avoid potential technological misapplications. This not only prevents time loss but also ensures effective reconfiguration. In turn, this dynamic capability-driven reconfiguration of workflows substantially mitigates the negative impact of teaching-research time conflict on GCQ.

Based on this theoretical derivation, the following hypotheses are proposed:

*H3a*: Challenge research stressors indirectly enhance GCQ by increasing AI literacy.

*H3b*: Hindrance research stressors indirectly diminish GCQ by decreasing AI literacy.

*H3c*: AI literacy buffers the negative effect of teaching-research time conflict on GCQ.

**Figure 1:**
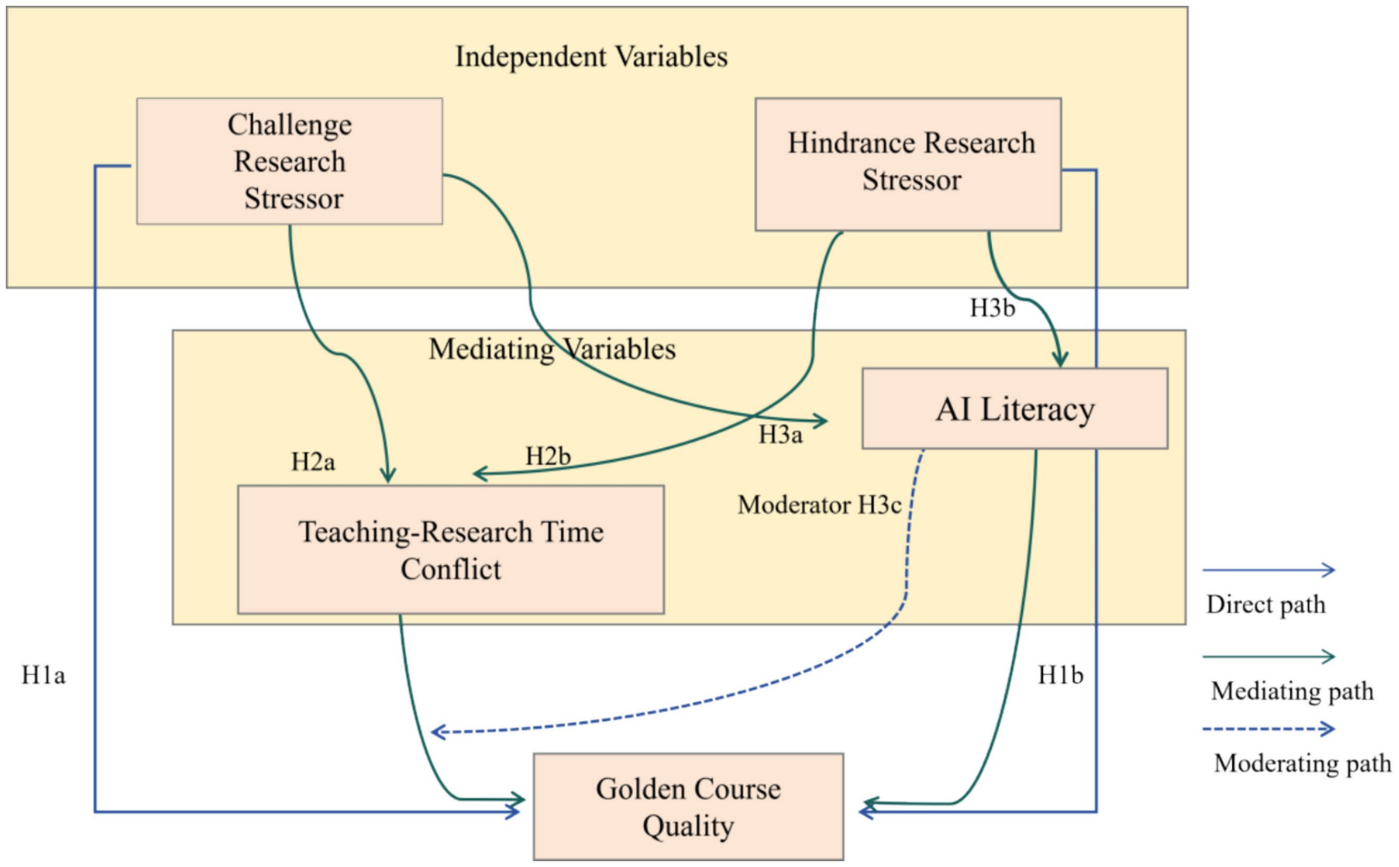
Proposed research model. Challenge and hindrance research stressors serve as independent variables. Teaching-research time conflict and AI literacy act as mediators. AI literacy also moderates the path from teaching-research time conflict to golden course quality. Arrows denote the hypothesized relationships. Challenge research stressors positively impact golden course quality (H1a). Hindrance research stressors may negatively impact golden course quality (H1b). Teaching-research time conflict mediates both stressors’ effects (H2a, H2b). AI literacy mediates the model pathways (H3a, H3b). Additionally, AI literacy buffers the impact of teaching-research time conflict on golden course quality (H3c).

Figure 1 presents the overall theoretical framework of this study. This framework incorporates all the aforementioned hypotheses that address the relationships among research stressors, AI literacy, teaching-research time conflict, and GCQ.

## 3 Methodology

### 3.1 Participants and procedure

This study used a stratified convenience sampling approach. Faculty participants were recruited from comprehensive academic universities in China, including those affiliated with the Project 985 and Project 211 initiatives, as well as general undergraduate institutions. Unlike vocational colleges, comprehensive universities typically impose dual pressures on faculty, requiring them to excel in both research output and teaching innovation. Accordingly, the tension between research expectations and the enhancement of teaching quality is particularly salient. This inherent tension renders the faculty an ideal sample for addressing our research questions.

To ensure sample diversity, we employed stratification based on three criteria. The first criterion was disciplinary field (STEM versus humanities and social sciences). The second was academic rank (from junior to senior positions). The third was institution type (general universities versus 211/985 universities). Questionnaires were distributed through professional faculty networks to instructors who engaged in both research and teaching. We collected 263 responses. After data cleaning, 253 valid samples were retained for analysis. Data cleaning excluded responses with completion times below 100 s and cases containing incomplete or missing entries. All participants met the essential criterion of being actively engaged in both research and teaching. Table 1 presents the demographic characteristics of the participating faculty.

**Table 1:** Profile of the participant teachers.

Group	Subgroup	N	%
Gender	Male	136	53.8
	Female	117	46.2
Age	≤30 years old	19	7.5
	31–40 years old	174	68.8
	41–50 years old	51	20.2
	≥51 years old	9	3.6
Education background	Bachelor	6	2.4
	Master	85	33.6
	Doctoral	141	55.7
	Postdoctoral	21	8.3
Title	Teaching assistant	35	13.8
	Lecturer	135	53.4
	Associate professor	67	26.5
	Professor	16	6.3
Subject	Science and Engineering	114	45.1
	Humanities and Social Sciences	100	39.5
	Medical Sciences	28	11.1
	Interdisciplinary disciplines	8	3.2
	Arts and Sports	3	1.2
Teaching age	≤5 years	103	40.7
	6–10 years	76	30.0
	11–15 years	45	17.8
	≥16 years	29	11.5
Type	Project 985 universities	14	5.5
	Project 211 universities	26	10.3
	General undergraduate universities	213	84.2

### 3.2 Measurement tool

Measurement items for latent variables were rigorously constructed based on a synthesis of established theoretical frameworks and empirical precedents. This approach ensured both conceptual validity and methodological robustness. All constructs used a five-point Likert scale (1 = strongly disagree, 5 = strongly agree). Table 2 provides the complete descriptions of all measurement items. We assessed challenge research stress and hindrance research stress using adapted scales comprising six and eight items, respectively (Zhang and Liao, 2014). For AI literacy, we adopted eight core items from Imjai et al. (2025), with two items per sub-dimension. This design comprehensively captured the hierarchical construct. Teaching-research time conflict was measured with a four-item scale modified from Carlson et al. (2000).

**Table 2:** Measurement model results for first- and second-order constructs.

Construct and items	Loadings
Challenge Research Stressor (1st-order reflective) (Cα = 0.805; CR = 0.862; AVE = 0.622)	
I often experience significant time pressure in my research work	0.789
My research work requires me to take on important responsibilities	0.798
Research work demands high-level and complex skills	0.711
I am expected to complete a heavy workload in my research	0.851
Hindrance Research Stressor (1st-order reflective) (Cα = 0.833; CR = 0.851; AVE = 0.595)	
I feel uncertain about how to become an outstanding scholar	0.728
I find it difficult to balance the dual roles of being an effective teacher and a productive scholar	0.818
My academic skills and capabilities seem to have plateaued	0.757
I experience a persistent lack of job security in my academic career	0.791
I have inadequate resources to conduct my academic research	0.759
Teaching-Research Time Conflict (1st-order reflective) (Cα = 0.913; CR = 0.918; AVE = 0.794)	
My research work occupies a significant amount of time, making it difficult to devote sufficient effort to teaching	0.836
I often sacrifice lesson preparation time to meet urgent research project deadlines	0.931
Urgent demands from research frequently force me to cancel or postpone teaching preparation plans	0.896
Time pressures from research projects prevent me from fully designing innovative teaching activities	0.899
AI Literacy (2nd-order reflective) (Cα = 0.939; CR = 0.942; AVE = 0.802)	
AI Basic (1st-order reflective) (Cα = 0.855; CR = 0.857; AVE = 0.883)	
I understand the basic concepts and principles of generative AI	0.930
I can clearly explain how generative AI systems work to others	0.939
AI Proficiency (1st-order reflective) (Cα = 0.805; CR = 0.812; AVE = 0.836)	
I can effectively design or optimize prompts for generative AI according to different task requirements	0.924
I frequently use generative AI in my study or work	0.904
AI Insight (1st-order reflective) (Cα = 0.862; CR = 0.863; AVE = 0.878)	
I have a deep understanding of the limitations and potential risks of using generative AI	0.934
I can effectively use generative AI to explore or develop new ideas and approaches in teaching or research	0.940
AI Analysis Capability (1st-order reflective) (Cα = 0.901; CR = 0.902; AVE = 0.910)	
I am capable of using generative AI to analyze complex problems and propose feasible solutions	0.956
I am able to use generative AI to predict trends or outcomes	0.952
Golden Course Quality (2nd-order reflective) (Cα = 0.782; CR = 0.831; AVE = 0.622)	
High-Order Thinking (1st-order reflective) (Cα = 0.732; CR = 0.733; AVE = 0.789)	
My course objectives are designed to integrate knowledge, skills, and competencies organically	0.884
My course content includes problem-solving-oriented tasks	0.892
Innovation (1st-order reflective) (Cα = 0.655; CR = 0.656; AVE = 0.743)	
Over 30% of my course content is updated annually (e.g., with new industry reports or research findings).	0.855
I employ digital tools (e.g., virtual labs, online collaboration platforms) or blended learning models in my teaching	0.869
Challenge level (1st-order reflective) (Cα = 0.738; CR = 0.743; AVE = 0.792)	
My course assessments include high-difficulty tasks	0.902
In my course, the ratio of in-class to out-of-class learning time is ≥1:2	0.877

Cα, Cronbach’s alpha; CR, composite reliability; AVE, average variance extracted.

GCQ was operationalized into three sub-dimensions that aligned with the “high-order thinking, innovation, challenge” framework. Higher-order thinking was measured using two items grounded in deep learning and experiential learning theories (Biggs, 1999; Entwistle and Ramsden, 2015; Kolb, 2014). Innovation was assessed using two items derived from the technological pedagogical content knowledge framework and curriculum renewal (Koehler and Mishra, 2009; Fullan, 2005). The learning challenge was measured using two items based on cognitive load theory and deliberate practice theory (Sweller, 1988; Ericsson et al., 1993). A panel of three higher education researchers with expertise in curriculum studies reviewed all measurement items to ensure content relevance and clarity. While using two-item subscales limits the estimation of internal consistency, this design prioritizes content validity. It ensures that the items faithfully reflect the construct as conceptualized in both the policy framework and the underlying theoretical foundations.

### 3.3 Common method bias

Data were collected via a single survey instrument, which raises the potential for common method bias (Podsakoff et al., 2003). To procedurally mitigate this risk, we implemented several procedural design safeguards: (a) psychological and temporal separation by presenting predictor, mediator, moderator, and outcome variables in distinct, unlabeled blocks; (b) ensuring respondent anonymity and emphasizing the absence of right or wrong answers to reduce social desirability bias; and (c) using varied scale formats and incorporating reverse-worded items where deemed appropriate. Statistically, we applied Harman’s single-factor test and a full collinearity assessment (VIF). The unrotated solution yielded a single factor accounting for 27.88% of the total variance, which is well below the 50% threshold. All construct-level VIF values were below 3.3, suggesting that neither severe multicollinearity nor method variance constitutes a dominant driver of the results.

### 3.4 Data analysis

This study employed partial least squares structural equation modeling (PLS-SEM) for data analysis. A key consideration is that our model integrates both confirmatory and exploratory elements. The core relationships involving challenge stressors (H1a and H3a) and the buffering role of AI literacy (H3c) are grounded in established theoretical frameworks. In contrast, the relationships involving hindrance stressors (H1b and H3b) are theoretically less certain and thus treated as exploratory. PLS-SEM is well-suited for such hybrid models. It accommodates both theory testing and theory development (Hair et al., 2019). Covariance-based SEM is primarily designed for confirmatory analysis (Reinartz et al., 2009). Another consideration is the complexity of our proposed model, which incorporates two second-order constructs (AI literacy and GCQ). The model also includes one moderating effect. PLS-SEM offers greater flexibility in estimating complex models, particularly when using the repeated indicators approach for higher-order constructs (Becker et al., 2012; Sarstedt et al., 2019). With respect to sample size, our sample of 253 exceeds the minimum threshold for covariance-based SEM in certain contexts (Reinartz et al., 2009). However, PLS-SEM offers superior statistical power for detecting effects in moderately complex models. These advantages are particularly clear when PLS-SEM is combined with nonparametric bootstrapping (Hair et al., 2019; Kock and Hadaya, 2018).

The analysis followed a two-stage PLS-SEM framework using SmartPLS 4.1.1.2. First, the measurement model was evaluated for reliability and validity. Second, the structural model was examined to test the study’s hypotheses regarding direct, indirect, and moderating effects, with 5,000 bootstrap iterations ensuring parameter stability. These methodological choices, consistent with PLS-SEM practices in educational technology research (Abbas et al., 2024) and recent strategic and management studies (Badulescu et al., 2025), ensure rigorous hypothesis testing while accounting for the study’s exploratory components.

## 4 Empirical results

### 4.1 Measurement model assessment

This study developed a two-stage analytical procedure. First, the measurement model was evaluated to ensure the reliability and validity of all constructs. These properties were assessed using indicators such as standardized factor loadings, Cronbach’s *α*, composite reliability (CR), and average variance extracted (AVE) (Hair et al., 2019). The structural model was then tested to verify the hypothesized relationships among the constructs.

**Figure 2:**
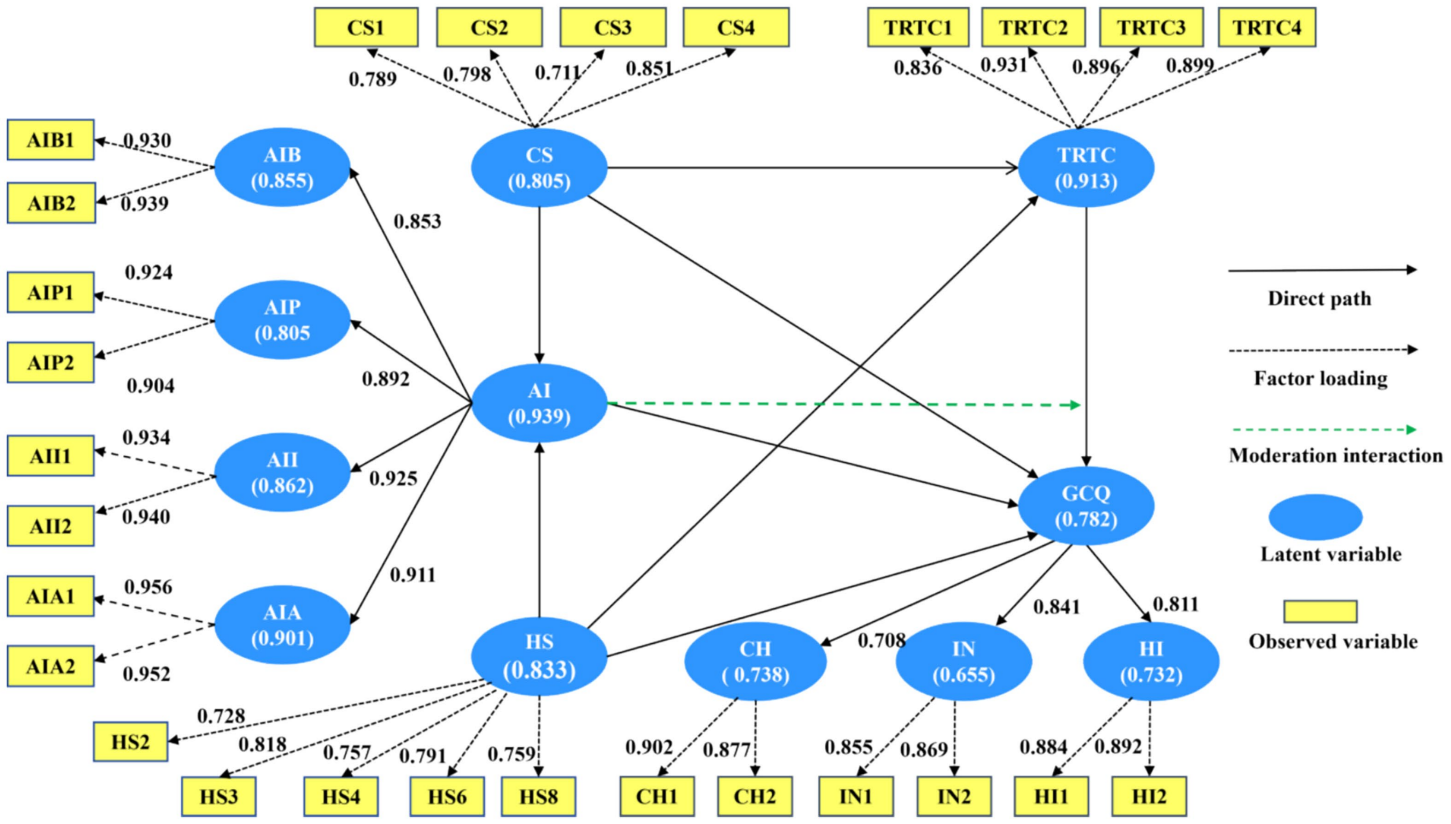
Measurement model. CS, challenge research stressors; HS, hindrance research stressors; TRTC, teaching-research time conflict; AI, AI literacy; GCQ, golden course quality; AIB, AI basic; AIP, AI proficiency; AII, AI insight; AIA, AI analytical capability. Standardized factor loadings are shown next to each arrow; all exceed 0.70, indicating strong item reliability. Cronbach’s alpha values are shown within each ellipse; values range from 0.655 to 0.939, all within acceptable limits for exploratory research. These results confirm good item reliability and acceptable internal consistency of the measurement model.

As shown in Table 2, all factor loadings exceeded 0.70. Cronbach’s α values ranged from 0.655 to 0.939, with the majority exceeding 0.70. Note that the value of 0.655 is still acceptable in the context of exploratory research. All CR values surpassed 0.70, and all AVE values exceeded 0.50. Thus, the measurement model demonstrated good reliability and convergent validity. Confirmatory factor analysis supported the higher-order structure of the two second-order constructs, i.e., AI literacy and GCQ. Detailed results are presented in Figure 2 and Table 2.

Discriminant validity, which ensures that the latent constructs are empirically distinct, was evaluated via the Heterotrait-Monotrait (HTMT) ratio of correlations (Henseler et al., 2015). As shown in Table 3, all HTMT values were below the stringent threshold of 0.85, providing strong evidence for discriminant validity.

**Table 3:** Discriminant validity assessment using the Heterotrait-monotrait ratio.

Construct	Mean	SD	CS	HS	TRTC	AI	GCQ
CS	3.728	0.689					
HS	3.397	0.762	0.202				
TRTC	3.053	0.943	0.329	0.469			
AI	3.230	0.693	0.235	0.031	0.247		
GCQ	3.326	0.552	0.404	0.202	0.231	0.428	

CS, Challenge research stressors; HS, Hindrance research stressors; TRTC, Teaching-research time conflict; AI, AI literacy; GCQ, Golden course quality.

Finally, to assess potential multicollinearity among the indicators, the VIFs were examined. The results reveal that all VIF values were well below the recommended limit of 5 (Hair et al., 2019), indicating that multicollinearity is not a concern in this study.

### 4.2 Structural model assessment

**Figure 3:**
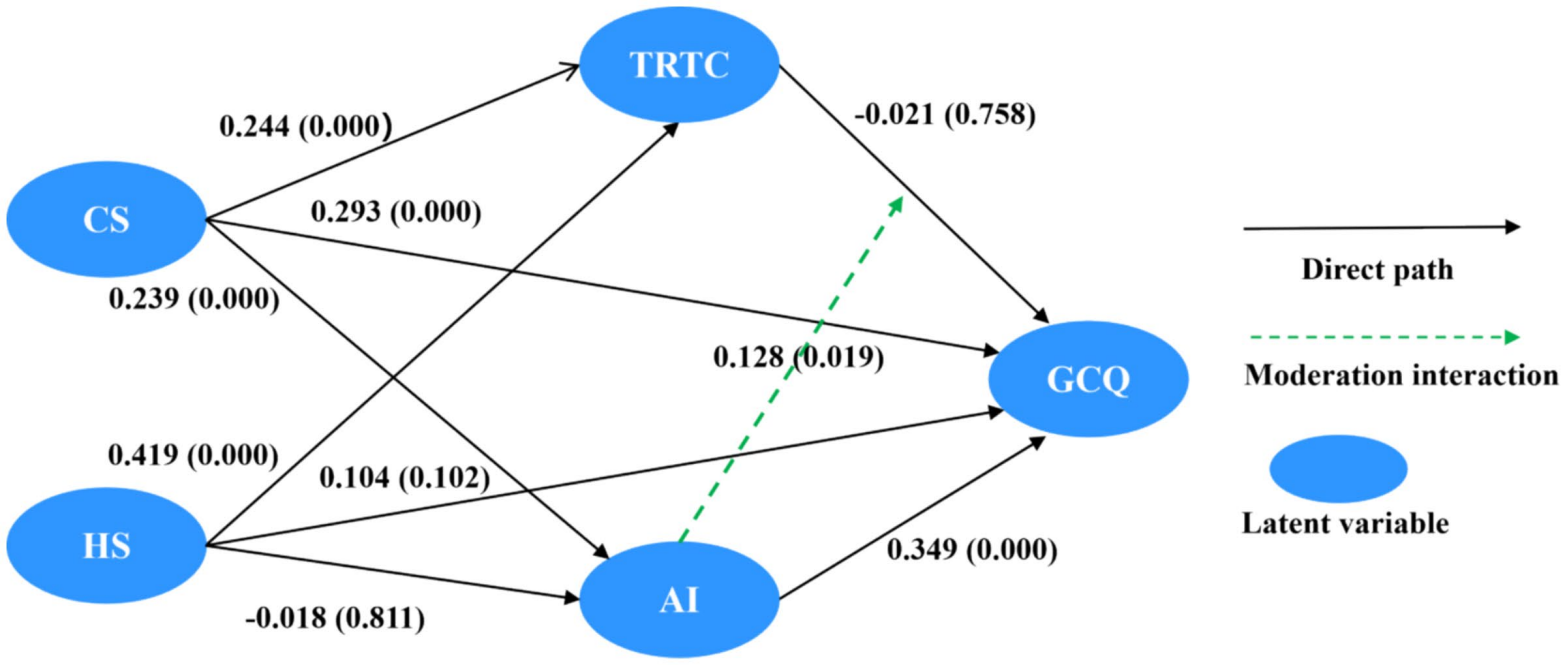
Structural model results with standardized path coefficients. CS, challenge research stressors; HS, hindrance research stressors; TRTC, teaching-research time conflict; AI, AI literacy; GCQ, golden course quality. Standardized path coefficients (*β*) are shown on lines, with *p*-values in parentheses. Key findings: CS → GCQ (β = 0.293, *p* < 0.001) supports H1a; HS → GCQ (β = 0.104, *p* = 0.102) does not support H1b; CS → TRTC (β = 0.244, *p* < 0.001) and HS → TRTC (β = 0.419, *p* < 0.001) are significant, but TRTC → GCQ (β = −0.021, *p* = 0.758) is not, thus H2a and H2b are rejected. CS → AI (β = 0.239, *p* < 0.001) and AI → GCQ (β = 0.349, *p* < 0.001) support H3a. HS → AI (β = −0.018, *p* = 0.811) is not significant, rejecting H3b. The interaction AI × TRTC → GCQ (β = 0.128, *p* < 0.05) supports H3c.

The study’s hypotheses concerning direct, indirect, and moderating effects were examined using bootstrapping procedures with 5,000 resamples in SmartPLS (Hair et al., 2019). The structural model is presented in Figure 3, and the detailed results of the hypothesis tests are summarized in Table 4.

**Table 4:** Hypotheses results.

Hypotheses	Relationships			β [T-values]	*p*-values	BCILL, BCIUL	Results
	IV	MV	DV				
Direct effects							
H1a	CS	→	GCQ	0.293 [4.598]	0.000	[0.166, 0.418]	Supported
H1b	HS	→	GCQ	0.104 [1.635]	0.102	[−0.026, 0.226]	Rejected
Mediating effects							
H2a	CS	→TRTC→	GCQ	−0.005 [0.295]	0.768	[−0.043, 0.028]	Rejected
H2b	HS	→TRTC→	GCQ	−0.009 [0.300]	0.764	[−0.069, 0.049]	Rejected
H3a	CS	→ AI →	GCQ	0.083 [2.735]	0.006	[0.033, 0.150]	Supported
H3b	HS	→ AI →	GCQ	−0.006 [0.234]	0.815	[−0.055, 0.050]	Rejected
Moderating effect							
H3c	AI	×TRTC	GCQ	0.128 [2.344]	0.019	[0.020, 0.233]	Supported

CS, Challenge research stressors; HS, Hindrance research stressors; TRTC, Teaching-research time conflict; AI, AI literacy; GCQ, Golden course quality.

First, the direct relationships among the constructs were assessed. The results in Table 4 demonstrate that challenge research stress exhibits a significant positive direct effect on GCQ (*β* = 0.293, *p* < 0.001), thereby supporting hypothesis H1a. By contrast, the direct effect of hindrance research stress on GCQ was not statistically significant (*β* = 0.104, *p* = 0.102). Consequently, hypothesis H1b, which posited a significant negative direct effect, is not supported.

Next, the hypothesized mediating role of teaching-research time conflict was examined. Both challenge research stress (*β* = 0.244, *p* < 0.001) and hindrance research stress (*β* = 0.419, *p* < 0.001) were found to have significant positive effects on teaching-research time conflict. However, the path from teaching-research time conflict to GCQ was negative but non-significant (*β* = −0.021, *p* = 0.758). The bootstrapping analysis for specific indirect effects (Preacher and Hayes, 2008; Hair et al., 2019) confirmed the absence of significant mediation: The specific indirect effect of challenge research stress on GCQ via teaching-research time conflict was *β* = −0.005, 95% CI [−0.043, 0.028] (containing zero), *p* = 0.768. Similarly, the specific indirect effect of hindrance research stress on GCQ via the teaching-research time conflict was β = −0.009, 95% CI [−0.069, 0.049] (containing zero), *p* = 0.764. Therefore, hypotheses H2a and H2b are rejected.

Regarding the mediating role of AI literacy, challenge research stress showed a significant positive impact on AI literacy (*β* = 0.239, *p* < 0.001). Conversely, hindrance research stress had a non-significant negative effect on AI literacy (*β* = −0.018, *p* = 0.811). AI literacy demonstrated a strong, significant positive effect on GCQ (*β* = 0.349, *p* < 0.001). The bootstrapping test for specific indirect effects revealed a significant specific indirect effect of challenge research stress on GCQ via AI literacy (*β* = 0.083, 95% CI [0.033, 0.150], *p* < 0.01). This finding supports hypothesis H3a. The specific indirect effect of hindrance research stress on GCQ via AI literacy was insignificant (*β* = −0.006, 95% CI [−0.055, 0.050] (containing zero), *p* = 0.081). Therefore, hypothesis H3b is rejected.

Finally, hypothesis H3c posited that AI literacy buffers the adverse impact of teaching-research time conflict on GCQ. The results indicated a significant positive effect of the interaction term (AI literacy × teaching-research time conflict) on GCQ (*β* = 0.128, *p* < 0.05). This significant positive interaction suggests that higher levels of AI literacy attenuate the negative association between teaching-research time conflict and GCQ, thereby functioning as a buffer. Consequently, hypothesis H3c is supported.

## 5 Discussion

This study integrated the COR Theory, the Challenge-Hindrance Stressors Framework, and the Dynamic Capabilities Theory to assess the dual-path effects of research stressors on GCQ and clarify the pivotal role of AI literacy. The results reveal asymmetric effects of challenge and hindrance stressors and provide empirical evidence for a technology-enabled mechanism that facilitates sustainable educational development.

Challenge research stressors exerted a significant positive influence on GCQ, thereby supporting Hypothesis H1a. One possible explanation is that challenging research tasks enhance complex problem-solving capabilities, interdisciplinary research methodologies, and critical thinking skills. These enhanced capabilities can be transferred into teaching practices through knowledge transfer mechanisms. This transfer directly elevates the core attributes of GCQ, namely, high-order thinking, innovation, and challenge. This suggests that when faculty perceive challenge stressors as opportunities for professional development, such pressure can be effectively transformed into a driver for teaching innovation. This finding extends the conclusion of Pitafi et al. (2025) that “goal-oriented stress promotes knowledge transfer to optimize innovation.”

Hindrance research stressors exerted a non-significant effect on GCQ, leading to the rejection of Hypothesis H1b. This finding does not align with the traditional Challenge-Hindrance Stressors Framework prediction of negative direct effects (Cavanaugh et al., 2000). However, it does not contradict the observation that hindrance stressors intensified the teaching-research time conflict. The resource depletion pathway was activated, but this activation did not translate into diminished teaching quality. One plausible explanation lies in the institutional valorization of teaching itself. Within the Chinese context, the “Golden Courses” policy explicitly designates teaching innovation as a strategic priority. It may strengthen the faculty’s commitment to safeguarding teaching quality, even when they face hindrance stressors. Identity-based mechanisms (McCune, 2019) and value hybridization strategies (De Graaf, 2021) may also contribute to this phenomenon, though these remain speculative without direct measurement. Conceptually, this finding refines the Challenge-Hindrance Stressors Framework by demonstrating that the direct negative effects of hindrance stressors on teaching outcomes are not deterministic. These effects depend on whether the outcome domain is institutionally valorized and whether faculty have access to buffering resources beyond the traditional time-based resource pool.

Notably, the mediating role of teaching-research time conflict was not supported, leading to the rejection of H2a and H2b. Although both stressor types intensified the teaching-research time conflict, neither significantly affected GCQ. This finding reveals a boundary condition for COR Theory by demonstrating that the classical mechanism linking resource loss to performance outcomes may be disrupted when faculty have access to technologies that enable resource reconfiguration. One explanation concerns generative AI’s ability to compress time spent on teaching preparation. Recent experimental evidence from a randomized controlled trial found that ChatGPT reduced lesson planning time by approximately 31% while maintaining instructional quality (Roy et al., 2024). By automating routine tasks, such as content generation, assessment design, and personalized feedback, AI tools enable faculty to accomplish more within fixed time constraints (Mulyani et al., 2025; Chiu, 2023). The buffering effect of AI literacy documented in this study is consistent with this interpretation, as faculty with higher AI literacy appear better positioned to leverage such efficiency gains when time is scarce.

A second explanation relates to measurement considerations. Our four-item measure of the teaching-research time conflict was adapted from Carlson et al. (2000) and primarily captures the quantitative dimension of time allocation. That is, the time each role consumes. It may not fully capture qualitative aspects. These include cognitive interference between roles, perceived control over one’s schedule, or the psychological toll of juggling competing demands.

A third possibility concerns the heterogeneity of teaching activities themselves. Li and Yang (2024) distinguished between curriculum-based teaching and research-oriented teaching. Curriculum-based teaching involves routine course delivery, whereas research-oriented teaching integrates cutting-edge research into pedagogy. They found that curriculum-based teaching time was negatively correlated with research output. In contrast, research-oriented teaching time positively promoted research productivity. By extension, time spent on research-oriented teaching activities may enhance rather than undermine teaching quality, even under conditions of time scarcity. This distinction aligns with our conceptualization of GCQ, which emphasizes innovation and integration of research.

The significant role of AI literacy further reflects technology’s reconfiguration of academic practice, effectively embodying a form of dynamic capability (Teece, 2007). On the one hand, challenge stressors indirectly enhanced GCQ by improving AI literacy, supporting H3a. Engaging with academic challenges drives faculty to develop and refine AI-related competencies, constituting the ‘seizing’ dimension of dynamic capabilities by actively acquiring and integrating new technological skills. These enhanced capabilities enable the use of GenAI to design interdisciplinary cases and create adaptive and challenging tasks (Chiu, 2023; Mulyani et al., 2025). This process reflects the development of dynamic capability building, where faculty not only sense the need for change but also seize and reconfigure resources through AI integration.

On the other hand, AI literacy significantly buffered the negative effect of teaching-research time conflict on GCQ, supporting H3c. Faculty with high levels of AI literacy leverage these reconfigurative capacities to transform time conflicts into manageable constraints. They automate routine tasks, employ AI for learning diagnostics, and deploy chatbots for student inquiries (Javaid et al., 2023; Chiu, 2023). In doing so, they engage in continuous resource recombination and process optimization, both of which are hallmarks of a dynamic capability. This buffering effect is crucial for the sustainability of teaching quality. It allows faculty to maintain high standards under pressure. Thus, it directly contributes to quality education.

However, hindrance stressors did not significantly inhibit AI literacy, thereby rejecting H3b. An adaptive mechanism in technology-rich environments may explain this result. As generative AI becomes central to academic workflows, foundational AI proficiency has shifted from an optional skill to a job requirement. This mirrors the mandatory adoption of basic computing skills in the 1990s (Venkatesh et al., 2012). Faculty are now required to maintain a baseline level of AI literacy, irrespective of external pressures. This pattern aligns with the extended technology acceptance model, which suggests that when technology becomes institutional infrastructure, external factors lose their influence on skill adoption. Consequently, hindrance stressors no longer strongly affect foundational AI competency (Venkatesh and Davis, 2000).

### 5.1 Theoretical implications

This study advances the theoretical discussion on the research-teaching nexus in higher education. It offers three key contributions.

First, this study extends and contextualizes COR Theory by introducing a differentiated perspective on research stressors. Contrary to the prevailing view that research pressure unidirectionally erodes teaching quality (Bak and Kim, 2015), our findings demonstrate that challenge stressors directly enhance GCQ and generate indirect value through faculty AI literacy. This asymmetric effect aligns with organizational behavior research (LePine et al., 2005). To our knowledge, this is the first application of this asymmetric effect to teaching innovation. Importantly, hindrance stressors exacerbated the teaching-research time conflict. This conflict did not exert a significant negative impact on GCQ. This result identifies a boundary condition for COR Theory by revealing that the traditional link between resource loss and performance outcomes can be attenuated in digitally enriched academic environments.

Second, this study expands the application of Dynamic Capabilities Theory by conceptualizing AI literacy as a dual-function capability. Faculty AI literacy mediates the positive impact of challenge stressors on teaching excellence. It also buffers the negative effects of the teaching-research time conflict. This dual mechanism provides empirical evidence to support the view that AI literacy constitutes a dynamic capability. It enables resource reconfiguration under time and resource constraints. By showing how human-AI collaboration alleviates resource scarcity, our findings offer a new perspective on resilient teaching practices. These practices can withstand modern academic pressures.

Third, this integrated framework extends the traditional zero-sum view of the research-teaching relationship (Bak and Kim, 2015; Hacker and Dreifus, 2010; Hattie and Marsh, 1996; Marsh and Hattie, 2002). By showing that challenge-based research pressure can enrich teaching quality and that AI literacy can decouple the link between time conflict and performance outcomes, our findings provide empirical support for a model of synergistic reciprocity. This model suggests that research and teaching are not inherently in constant competition for limited resources; instead, they can reinforce each other under the right conditions, namely the presence of challenge-oriented stressors and dynamic technological capabilities. This offers a new theoretical blueprint for universities, suggesting that institutionalizing a research-teaching balance should not focus solely on managing resource trade-offs, but also on actively fostering positive challenge appraisals and enabling digital resource reconfiguration to support long-term sustainability.

### 5.2 Practical implications

Building on these findings, higher education administrators can adopt the following practical framework.

Higher education administrators should implement stress-governance strategies organized by category to stimulate research motivation and mitigate developmental barriers, thereby fostering a sustainable academic environment. For challenge stressors, establishing systematic translation mechanisms is essential. For instance, institutions can support faculty in transforming cutting-edge research outcomes into high-quality teaching resources. They can also incorporate the effectiveness of such translations into indicators for promotion and teaching performance evaluation. These practices encourage the integration of research knowledge into teaching. For hindrance stressors, interventions should focus on addressing their root causes. Potential measures include providing early-career faculty with streamlined professional development guidance, establishing micro-grants for interdisciplinary collaboration to alleviate resource constraints, prudently optimizing appraisal cycle design (e.g., piloting mid- to long-term evaluation mechanisms), and facilitating workshops on integrating teaching and research roles.

Given that AI literacy has been shown to have a dual enabling function, universities should prioritize its systematic development in their institutional strategies. On the one hand, AI literacy mediates the transformation of challenge stressors into GCQ; on the other hand, it buffers the potential erosive effect of teaching-research time conflict on teaching quality. We therefore recommend that universities integrate AI literacy cultivation into their strategic development agendas as a key investment to support educational sustainability.

Specifically, a tiered, needs-oriented training system should be developed. For faculty engaged in high-challenge research tasks, workshops focused on “GenAI-enabled teaching-research integration” should be designed. These workshops would cultivate their ability to transform complex research findings into intelligent learning tools. For faculty facing high hindrance stressors, training should prioritize the application of GenAI tools to address specific academic development challenges. Recent scholarship further suggests that generative AI does not inherently undermine academic integrity; its impact depends on how it is integrated (Tan and Maravilla, 2024). Clear institutional policies, AI literacy training that emphasizes critical evaluation of AI outputs, and assignment designs that value process and reflection can reframe AI from a potential risk to a valuable pedagogical asset. Promoting the establishment of an inter-institutional, discipline-specific repository of ethically reviewed AI teaching prompts can lower the barrier to technological application through resource sharing, particularly benefiting institutions with limited resources. This cultivates the resilient and innovative academic workforce central to achieving sustainable educational development.

To foster a symbiotic ecosystem for research and teaching, we propose adaptive optimizations within golden course certification systems. In national and provincial golden course evaluation frameworks, a new dimension, “research integration intensity,” could be introduced. This dimension would explicitly require course designs to articulate how cutting-edge research findings from the past 3 years have been translated into pedagogical elements that, in turn, enhance the course’s focus on high-order thinking, innovation, and challenge. It would provide practice-oriented institutional impetus for knowledge fusion and the continuous enhancement of education quality.

### 5.3 Limitations and future research directions

This study clarifies how research stressors influence GCQ in the era of generative AI. It also highlights the critical role of AI literacy. However, several limitations warrant further investigation.

First, the sample composition may limit generalizability. Our sample included various institutions, disciplines, and academic ranks. However, it contained a high proportion of faculty from standard undergraduate colleges. This imbalance may affect the applicability of our findings to elite research universities. When interpreting the research results, it is necessary to carefully consider the sample’s composition. Additionally, this study focused only on universities in mainland China, and its unique cultural and institutional background may affect the cross-cultural applicability of the results. Future studies should further test the model’s contextual boundaries through stratified sampling across different types of institutions and through cross-cultural comparative studies.

Second, measurement and methodological constraints should be noted. We implemented procedural controls, such as temporal separation and anonymity, and used Harman’s single-factor test and VIF diagnostics to assess the potential influence of common-method bias. However, these diagnostic techniques have well-known limitations. They cannot definitively rule out self-report inflation. This inflation may have affected the magnitude of the observed relationships. This concern is particularly acute for the outcome variable, GCQ. Faculty may overreport their teaching performance on socially valued dimensions, such as innovation and challenge. Consequently, the positive effects of challenge stressors and AI literacy on GCQ might be overestimated. The non-significant results for hindrance stressors and teaching-research time conflict could also be obscured or distorted by shared method variance. Readers should therefore interpret the effect sizes with caution. Future research should incorporate multi-source evaluations, such as student ratings, peer observations, and learning analytics, to triangulate self-reported teaching quality.

Furthermore, we used two-item measures for each GCQ dimension. While this approach ensured content validity, it sacrificed breadth of construct for parsimony. Reliability estimates were acceptable. However, shorter scales may not fully capture the construct’s scope. This limitation could affect effect sizes or null finding interpretations. Future research should develop and validate comprehensive GCQ scales. Additionally, AI literacy was operationalized as perceived competence, which may deviate from actual behaviors (Ng et al., 2021b). Finally, the cross-sectional design cannot capture dynamic changes over time. Rapid advancements in generative AI may also affect the timeliness of our conclusions. Future studies should adopt mixed methods. They should develop multi-indicator evaluation criteria for course quality. Researchers could also use log data to track actual AI usage patterns. Longitudinal designs would better map developmental trajectories. Living laboratory approaches could monitor emerging application paradigms in real time.

Third, contextual factors require deeper exploration. Several potential moderating effects have not been fully examined. These include disciplinary culture, perceived organizational support, and task-specific GenAI use. Such effects may differ between research and instructional tasks. For example, Xu and Shen (2025) found that STEM researchers use generative AI more frequently for research tasks than humanities scholars do. Perceived organizational support also moderates this difference. Future research should conduct discipline-stratified comparative analyses and incorporate variables such as organizational support and transformational leadership. Micro-level studies are also needed to clarify how generative AI functions across diverse academic tasks.

## Data Availability

The raw data supporting this article will be made available by the corresponding author upon reasonable request.
